# Domestic Use of the Exoskeleton for Gait Training in Patients with Spinal Cord Injuries: Ethical Dilemmas in Clinical Practice

**DOI:** 10.3389/fnins.2018.00078

**Published:** 2018-02-15

**Authors:** Luciano Bissolotti, Federico Nicoli, Mario Picozzi

**Affiliations:** ^1^Rehabilitation Service, Casa di Cura Domus Salutis, Fondazione Teresa Camplani, Brescia, Italy; ^2^Biotechnology and Life Sciences Department, Center for Clinical Ethics, Insubria University, Varese, Italy; ^3^Clinical Ethics Service, Domus Salutis Clinic, Teresa Camplani Foundation, Brescia, Italy

**Keywords:** exoskeleton spinal cord injury, ethics, Medical, financial, principlism, gait

## Introduction

In this paper, as in our previous works^1^ we evaluate some ethical questions about the domestic use of the robotic exoskeleton (ReWalk Robotics, Marlborough, MA, USA) (Esquenazi et al., 2012; Asselin et al., 2016) for gait assistance in people affected by a Spinal Cord Injury (SCI) (National spinal cord injury statistical center, 2010; Scivoletto et al., 2011). This device is presently FDA and EC market approved and it is now available in two different versions, one for hospital training and one for home-based use. The latter can be provided to the patient when a sufficient level of competence has been reached after special training. This work focuses on this second version of the ReWalk, since it was designed exclusively for domestic use.

Ethical concerns may arise because financial coverage of the home ReWalk version is still under debate for most patients; it depends mainly on personal resources in so far as home delivery is not supported by common and shared International Provisional rules.

In addition, in SCI patients the long term global consequences for health are marked by an increased risk of cardiovascular and metabolic diseases, while the paretic limbs may have a high risk of osteoporosis, skin lesions and deep venous thrombosis. Bowel constipation and pelvic floor impairment are other negative effects of SCI.

Patents with SCI, their relatives, and their health care providers frequently classify the recovery of the ability to walk as a high priority, even where great effort has been made to alleviate the aforementioned consequences (American Spinal Cord Injury Association, 1982; Nene et al., 1996).

To counteract these negative effects, Gait Retraining Programs have been operating for many years in order to exploit body-weight-supported gait on a treadmill (Sale et al., 2012), dynamic orthoses based upon passive mechanical hip-knee-ankle-foot orthoses (H-KAFO) which enable patients with SCI to ambulate over ground (Massucci et al., 1998) and/or similar synergic actions of Functional Electrical Stimulation (FES) with synchronized activation according to the different phases of the gait cycle (Nene and Patrick, 1990). Unfortunately, this type of treatment only results in obtaining a very slow gait speed with a high fatigue component.

Given these limitations, over the past 6 years the branch of Bioengineering applied to SCI rehabilitation has progressively introduced a few powered exoskeletons to induce robotic gait in AIS A SCI patients (Chen et al., 2013). A recent systematic review of the literature supports the effectiveness of different types of exoskeletons used in a rehabilitative setting in improving some physical conditions such as spasticity and blood pressure or certain aptitudes such as balance and autonomy in transfer (Esquenazi et al., 2012).

## Scientific evidence

According to data published in the literature, training with ReWalk can be provided only once specific inclusions criteria are satisfied: patients must be 18–65 years old, with a spinal cord injury between C6 and T12, and a score on the Modified Ashworth Scale equal to or less than 3, or absence of any joint constraints as determined by clinicians (Asselin et al., 2016).

As described in a recent meta-analysis conducted by Miller et al. (2016), it is interesting to note that effectiveness and safety of exoskeletons in SCI patients appear to be well consolidated in the International scenario. In this meta-analysis including 111 patients from 14 studies (8 of them analyzed the use of ReWalk), 76% of the patients were able to walk independently with the exoskeleton at the end of the treatment period and gait speed-averaged 0.28 m/s (range: 0.031–0.71 m/s). This review also showed a reduction of spasticity in 38% of the cases and an improvement of bowel management in 61%, while the metabolic demand averaged 3.3METs at a Borg mean value of 10 in a scale from 6 to 20 points. In this review negative side effects were represented by falls (4.4%) and bone fractures (3.4%). Even though this type of technology has not proved to be effective in enhancing autonomous gait recovery in SCI patients, it can be considered both as an assistive device to realize independent assisted walking and a rehabilitation tool for its ability to provide effective benefits to specific functions (i.e., bowel movements) and structures (i.e., trunk control) of the human body. Powered exoskeletons can be considered as motorized orthoses and prescribed as devices for domestic use with the purpose of allowing standing, walking, climbing stairs, and performing activities of daily living in a standing position.

Other Authors provided the evidence of improvement in some aspects of Quality of Life (General Health and the Physical Role of the SF12) and a good overall level of satisfaction with the use of ReWalk (Platz et al., 2016).

After a selection of participants, a step-by-step procedure introduced the patients to the following phases of the program (Asselin et al., 2016). The seven different steps are represented by: Fitting (the exoskeleton is adjusted according to the patient's measures); Donning (the specific strategy used to get into the ReWalk is explained); Standing (patients receive instructions about the way to control the activation of the exoskeleton while using crutches to stand up); Standing balance (while standing, shifting the body weight and moving the trunk to induce the walking phase); Walking with crutches and progressive goals in mobility (turning by 90 and 180°, arresting gait, walking through doors, using an elevator and walking close to other people); Sitting and Doffing.

All the procedures take place while a trained instructor is assisting the patient in all the different phases of the training program. The level of intervention is progressively reduced as the patient accomplishes all of the required functional tasks.

## Should i respond to the budget or to the person?

Financial coverage for domestic use of the ReWalk is still not supported by common and shared national and international provisions. Concerns of an ethical nature may arise since the domestic use of the device depends mainly on personal resources.

Thus in hospital settings the use of the exoskeleton for therapeutic purposes seems to be ethically justified because one single device can be available to many different patients for a limited period of time, and specifically to individualized therapeutic targets (Platz et al., 2016; Raab et al., 2016). This instrument appears to be a proportionate treatment for those patients affected by SCI. But the excessive costs (around $70,000.00) seem to influence the purchase for home use: “currently the technology for enhancement of the disabled is somewhat costly, limiting access to those few who can either afford to purchase access to the technology or those lucky enough to have health insurance plans that will pay for the costs associated with using this technology” (Greenbaum, 2015).

Good medical reasoning balances risks and benefits, and the use of the exoskeleton entails many of benefits. But the ethics issue, and not only the clinical issue, concerns what type of information the physician should offer the patient (Deledda et al., 2013). It also concerns resolving an ethical doubt: is it more correct to inform the patient, who will certainly improve in any case, but not as he would if he had the tool at home, of these facts? Or would it be more ethically correct to choose not to inform the patient a priori about this possible improvement in performance using the device at home unless he is the one who specifically asks (and presumably has the financial resources to pay for it)?

Another ethically relevant question regards the content of the information that is offered to the patient by the physician—even when patients can receive information from other sources—about both the possibility of using this instrument in a hospital, and at the same time, regarding the difficulty of achieving more effective improvements where there is no continued use of the instrument at home. In particular, the initial proposal to use the exoskeleton at home should not come from the patient, even if he/she already knows this device.

Where real economic difficulties exist: should the physician recommend this device a priori also as a “tool for domestic use”? Or, should he/she present it only as an additional (but costly) possibility?

This question may be analyzed using the four-principle approach, or Principlism. It attempts to examine ethical aspects of a biomedical question. They derive from “considered judgment” (Beauchamp and Childress, 2009) in the common morality and daily activities of healthcare professionals. These principles, as presented by Beauchamp and Childress (Engelhardt, 1996), can be used to resolve bioethical controversies.

They are: Respect for autonomy (respecting the decision-making capacities of autonomous persons; enabling individuals to make rational informed choices); Non maleficence (avoiding the causation of harm; the harm should not be disproportionate to the benefits of treatment); Beneficence (it considers the balancing of the benefits of treatment against the risks and costs; the healthcare professional should act in a manner which benefits the patient); Justice (distributing benefits, risks and costs fairly; the notion that patients in similar positions should be treated in a similar manner).

## The domestic use of the exoskeleton involves all four of these principles

The patient's autonomy of choice should be always maintained, even in the case of refusal of treatment for economic reasons, and even if the patient should not be left alone in the decision-making process. The doctor - patient/family relationship should be considered the most convenient setting to make a free and conscious decision about specific treatments (Nelson, 2012). In particular, regarding the domestic use of the exoskeleton, the patient often needs both the doctor's and a family member's word. The physician is always called upon to take care of his/her patients, according to the criterion of the proportionality of the treatments (Nicoli and Picozzi, 2017), by helping the patient to understand the benefits and risks of treatment in order to ensure autonomous decision-making. Even the family members have a specific role in the domestic use of the exoskeleton, because the patient needs another person to put on and to use the device. An essential requisite is to clearly and precisely show the different outcomes of the use of the exoskeleton while respecting the patient's choice (Kaldjian et al., 2005; Geppert and Shelton, 2012).

In an essential good doctor-patient relationship, special attention is asked to be paid by the doctor to the possible emotional and psychosocial difficulties (following the principle of non-maleficence) that the patient might have to face knowing that his/her therapy would be more effective if continued at home, but that he does not have the possibility of buying the exoskeleton: “There may be concerns that the availability of exoskeletons will create a dependency on the technology, and a limited availability will lead to withdrawal-like symptoms, whereby disabled individuals who may have relied on the technology, may exhibit psychosocial withdrawal-like symptoms when they lose access, either because of scarcity of the product or because they can no longer afford access to it” (Platz et al., 2016).

The third principle, that of beneficence, responds to the most important aim of medicine: to provide benefits to others. In particular, as expressed in the Hippocratic oath: “to help, or at least to do no harm” (Ama Council on Ethical Judicial Affairs, 2012).

Over the past decades this principle has often been presented in contrast with the patient's right to choose autonomously about the treatments medicine has to offer: “the principle of respect for autonomy has grounded several rights for patients, including the right to receive information, to consent to and refuse procedures, and to have confidentiality and privacy maintained. Other ethicists ground such obligations on the health care professionals' primary obligation to beneficence, which means to act for the patient's medical benefit”^2^.

This argument finds its origins in the concept of *Paternalism*, an attitude according to which the physician is compared to a father and the patients to his children.

The distinction between soft and hard paternalism could better balance the principles of autonomy and beneficence. Hard paternalism seems not to consider the patient's opinion about his/her own choices, and the physician is thought of as the only well-informed individual able to perform care-planning for the patient's good. Soft paternalism seems resolve the conflict between the principle of beneficence and the principle of autonomy: it reflects “the intended beneficiary's own conception of his/her best interest, even if the intended beneficiary fails to fully understand or recognize those interests or to fully pursue them because of inadequate willfulness, commitment, or self-control”^3^. This conception of soft paternalism seems to be able to respect the autonomy of the patient, because it is oriented toward offering support so that the patient can choose the best solution for him/herself.

This is important in order to legitimize both the choice of the doctor who proposes the exoskeleton, and, the patient's option of refusing it.

The principle of justice is the last step involved in solving the ethical dilemma about the use of this device in a patient with economic difficulties. In its contemporary form, this principle is sometimes expressed as follows: “Individuals should be treated in the same manner, unless they differ in ways that are relevant to the situation in which they are involved” (Velasquez et al., 1990).

Indeed, Beauchamp and Childress underscore certain difficulties concerning the principle of justice regarding *allocating, rationing* and *setting priorities*.

The third aspect, the setting of priorities, is dedicated to costs, especially for insurance, new technology, deteriorating health conditions and longer life expectancy: “the question in setting priorities is how to determine what ought to be done when resources are inadequate to provide all of the health benefits that it is technically possible to provide”^4^. Unfortunately inconsistent results regarding the effectiveness of the use of the Exoskeleton are reported in the literature. Therefore, studies with patients who are chosen very selectively are needed to demonstrate the efficiency and effectiveness of this device in order to offer it to a larger number of patients (Datteri, 2013; Iosa et al., 2016; Raab et al., 2016).

At home, the use of the exoskeleton might be evaluated as a good choice if the economic burdens do not weigh solely on the patient: the excessive cost of the device should not eliminate a priori this possibility for the single patient.

Offering the opportunity for domestic use means building preferential paths with associative realities or foundations (e.g. patients' associations) aimed at offering specific services to a vaster number of patients, so as to increase the therapeutic efficacy and to shorten the waiting list.

## Conclusion

In SCI patients, according to the available data, the use of exoskeletons is clinically and ethically justified. So far, the high cost of the device represents the main limiting factor for the adoption in home environments and places the issue of inequality on the access of technologically advanced assisted devices. This should not eliminate a priori this possibility for the single patient, since the health-care market is at present offering an array of solutions to various health-related problems which are based on patients' more or less limited economic possibilities.

To offer an opportunity for the domestic use of the instrument means to build preferential paths with patients' associations or foundations in order to offer specific services for a larger number of patients, so as to increase therapeutic efficacy and decrease inequality in the access to health care services and devices.

## Author contributions

LB developed the theoretical formalism for rehabilitation background and exoskeletons and performed the literature research. FN developed the theoretical formalism for ethics background and rehabilitation implications and performed the literature research for this area of interest. All three authors, LB, FN, and MP contributed to the final version of the manuscript. MP supervised the project.

### Conflict of interest statement

The authors declare that the research was conducted in the absence of any commercial or financial relationships that could be construed as a potential conflict of interest.

## Footnotes

^1^This case was initially presented at the 26th International Conference on Robotics in Alpe-Adria-Danube Region, L. Bissolotti, P. Zuccher, A. Zenorini, S. Chiari, P. Gaffurini, A. Pasini, F. Nicoli, Exoskeleton for Gait Training in Spinal Cord Injured people: clinical analysis and ethical Dilemmas, 2017 June 21-23 Tourin; and at the 31th European Conference on Philosophy of Medicine and Health Care (ESPMH), Exoskeleton for gait training in spinal cord injuried people: Should I respond to the budget or to the person?, Belgrade, Serbia 16-19 agosto 2017. A full version of this paper was accepted as chapter of the book “Advance in Service and Industrial Robotics” (eds. C.,Ferraresi, G. Quaglia) Springer, L., Bissolotti, P., Zuccher, A., Zenorini, S.,Chiari, P., Gaffurini, A Pasini, F. Nicoli. Exoskeleton for Gait Training in Spinal Cord Injuried People: Clinical Analysis and Ethical Dilemmas, 2018, pp. 759–765.

^2^Beauchamp, T. L., Childress, J. F. Principles of Biomedical Ethics. cit., 207.

^3^Beauchamp, T. L., Childress, J. F. Principles of Biomedical Ethics. cit., 210–211.

^4^Beauchamp, T. L., Childress, J. F. Principles of Biomedical Ethics, cit., 267–275.
